# Long-lasting severe anemia following treatment with natalizumab for relapsing–remitting multiple sclerosis: a case report

**DOI:** 10.1186/s13256-024-04562-8

**Published:** 2024-05-13

**Authors:** Lars Henrik Dahl Hamnvik, Geir E. Tjønnfjord, Signe Spetalen, Jakob Dalgaard

**Affiliations:** 1 Department of Medicine, Drammen Hospital, Vestre Viken Trust, Drammen, Norway; 2 Department of Haematology, Oslo University Hospital, Oslo, Norway; 3 Department of Pathology, Oslo University Hospital, Oslo, Norway; 4 Institute of Clinical Medicine, KG Jebsen Centre for B-Cell Malignancies, University of Oslo, Oslo, Norway

**Keywords:** Anemia, Natalizumab, Inhibited maturation, VLA-4, Multiple sclerosis

## Abstract

**Background:**

Natalizumab is a monoclonal antibody used to treat patients with relapsing–remitting multiple sclerosis. Anemia is a recognized side effect, but it is usually mild and of a short duration when natalizumab is stopped. Here, we describe a case of a young woman with severe and especially long lasting anemia associated with treatment with natalizumab, persisting up to a year after treatment was stopped.

**Case presentation:**

A 24 year-old Caucasian woman with relapsing–remitting multiple sclerosis developed severe transfusion dependent anemia after 27 infusions with natalizumab, which was her first and only treatment for her multiple sclerosis. Extensive hematologic diagnostics did not reveal any malignant cause or any other plausible non-malignant cause for her anemia. The bone marrow was found to be hypercellular, with a maturation arrest of the erythropoiesis and with grade 1–2 fibrosis. No specific treatment for the anemia was given. The hemoglobin level showed signs of spontaneous increase after nearly one year after natalizumab was discontinued.

**Conclusion:**

Severe anemia can be caused by treatment with natalizumab. This case adds information to the few other similar reported cases, demonstrating the potential duration of the anemia, as well as detailed description of hematologic findings. The mechanism is most likely due to inhibition of α4 subunit of the α4β1-integrin, which is present on both lymphocytes and erythroid precursor cells.

## Introduction

Natalizumab is a monoclonal antibody used to treat patients with relapsing–remitting multiple sclerosis (RRMS). The antibody selectively binds and inhibits the α4 subunit of the α4β1-integrin, very late antigen-4 (VLA-4), blocking interaction with vascular cell adhesion molecule 1 (VCAM-1) [1]. This inhibition prevents the transmigration of lymphocytes over the blood–brain barrier, resulting in reduced inflammation in the central nervous system. Natalizumab has a number of potential side effects, of which progressive multifocal leukoencephalopathy has gained much attention and awareness. Different hematologic side effects are known, and natalizumab has been reported to cause severe anemia [2–6]. Different putative mechanisms behind anemia may exist. Natalizumab has been shown to cause severe direct anti-globulin test (DAT) positive hemolytic anemia after only one or two infusions. More importantly, the blocking of α4β1-integrin can interrupt the communication between macrophages and erythroblasts, which is important for red cell maturation. Here, we describe another case of an unusually long-lasting severe anemia associated with treatment with natalizumab, most likely due to the last mentioned mechanism.

## Case

A 24-year-old Caucasian woman was diagnosed with RRMS 30 months before the present admission. At the time of the RRMS diagnosis, she had reduced vision and general hyperreflexia and suffered from fatigue. After two months of observation, treatment with monthly subcutaneous injections with natalizumab was initiated and subsequently considered well tolerated. Following 27 infusions of natalizumab the patient complained of headaches. One month later, severe anemia with a hemoglobin of 6.2 g/dL was detected, and the patient was admitted for hematological investigations. She had never received corticosteroids or any other form of MS treatment.

The patient complained of fatigue and dyspnea on activity over the past 2–4 months. With regard to potential blood loss, she had amenorrhea due to injections with medroxyprogesterone acetate every three months and fecal occult blood test was negative. A mild tachycardia of 110 was noted, and her mucous membranes were pale. On physical examination no enlarged lymph nodes were found and the liver and spleen was not palpable. There were no other clinical findings apart from her neurological disability.

Laboratory results revealed macrocytic anemia, mild neutropenia, and normal thrombocytes. Apart from increased lactate dehydrogenase and decreased haptoglobin, the laboratory parameters were within normal ranges (Table 1). Vitamin B9, B12 and metylmalonic acid were normal. She received transfusions with packed red cells. Viral serology indicated previous CMV and EBV infection, and antibodies for hepatitis B and C viruses were negative. A blood smear showed anisocytosis with macrocytosis. No spherocytes, schistocytes or red blood cell agglutination was detected. Thus, there were no morphological signs of hemolysis. A bone marrow smear displayed increased cellularity, with an adequate number of megakaryocytes without dysplastic features. Granulocytopoiesis was normal. Erythropoiesis constituted 35% of all nucleated cells, with a left shift indicated by a relative lack of polychromatic and pyknotic erythroblasts. There were no signs of inclusion bodies in erythroid precursors, as can be seen in parvovirus infection.

**Table 1:** Laboratory data at admittance

Variable	Results	Reference range (Adults)
C-reactive protein (mg/L)	8	0–5
Hemoglobin (g/dL)	6.2	11.7–15.3
Mean corpuscular volume (fL)	112	82–98
Mean corpuscular hemoglobin (pG)	37	27–33
Reticulocytes (× 10^9^/L)	30	30–100
Reticulocytes (%)	1.8	0.2–2.0
Reticulocytes hemoglobin concentration (pG)	40	28–35
Leukocytes (× 10^9^/L)	3.2	3.5–11.0
Neutrophils (× 10^9^/L)	1.33	1.5–7.0
Lymphocytes (× 10^9^/L)	1.53	0.8–3.7
Monocytes (× 10^9^/L)	0.23	0.2–0.8
Eosinophils (× 10^9^/L)	0.05	0.0–0.4
Basophils (× 10^9^/L)	0.02	0.0–0.1
Thrombocytes (× 10^9^/L)	259	145–390
Vitamin B_12_—holo-transcobalamin-2 (pmol /L)	44	> 31
Folic acid (nmol/L)	11.1	4.2–21.7
Methylmalonic acid (µmol L)	< 0.26	< 0.26
Ferritin (µg/L)	283	15–200
Transferrin (g/L)	1.9	2.0–3.3
Serum Iron (µmol/L)	44	9–34
Transferrin saturation (%)	94	10–50
Transferrin receptor (mg/L)	0.81	0.76–1.76
Creatinine (µmol/L)	81	45–90
eGFR (mL/min/1.73m^2^)	88	> 80
Lactate dehydrogenase (U/L)	338	105–205
Bilirubin (µmol/L)	16	5–25
Alanine aminotransferase (U/L)	26	10–45
Alkaline phosphatase (U/L)	76	35–105
Albumin (g/L)	39	36–48
Haptoglobin (g/L)	0.06	0.4–2.1
IgA (g/L)	2.0	0.7–4.3
IgG (g/L)	9.8	6.9–15.7
IgM (g/L)	1.17	0.6–2.3
Electrophoresis	No monoclonal protein detected	
Free kappa chain (mg/L)	22.7	6.7–22.4
Free lambda chain (mg/L)	28.3	8.3–27.0
Kappa/lambda ratio	0.8	0.31–1.56

*eGFR* Estimated glomerular filtration rate

Flow cytometry of the bone marrow showed a normal percentage and phenotype of CD34 + cells, a marked left shift in erythropoiesis, and a slight discordant expression of CD71 and CD36. A CT scan of the neck, thorax, abdomen, and pelvis demonstrated a slightly enlarged spleen with an axial diameter of 15 cm and a homogeneous density with no focal lesions. With a low haptoglobin, elevated lactate dehydrogenase, and splenomegaly, hemolytic anemia was considered; however, the reticulocyte count was not elevated, and bilirubin was normal. DAT was positive for anti-C3d and negative for anti-IgG. Cold agglutinins were not detected.

**Fig. 1:**
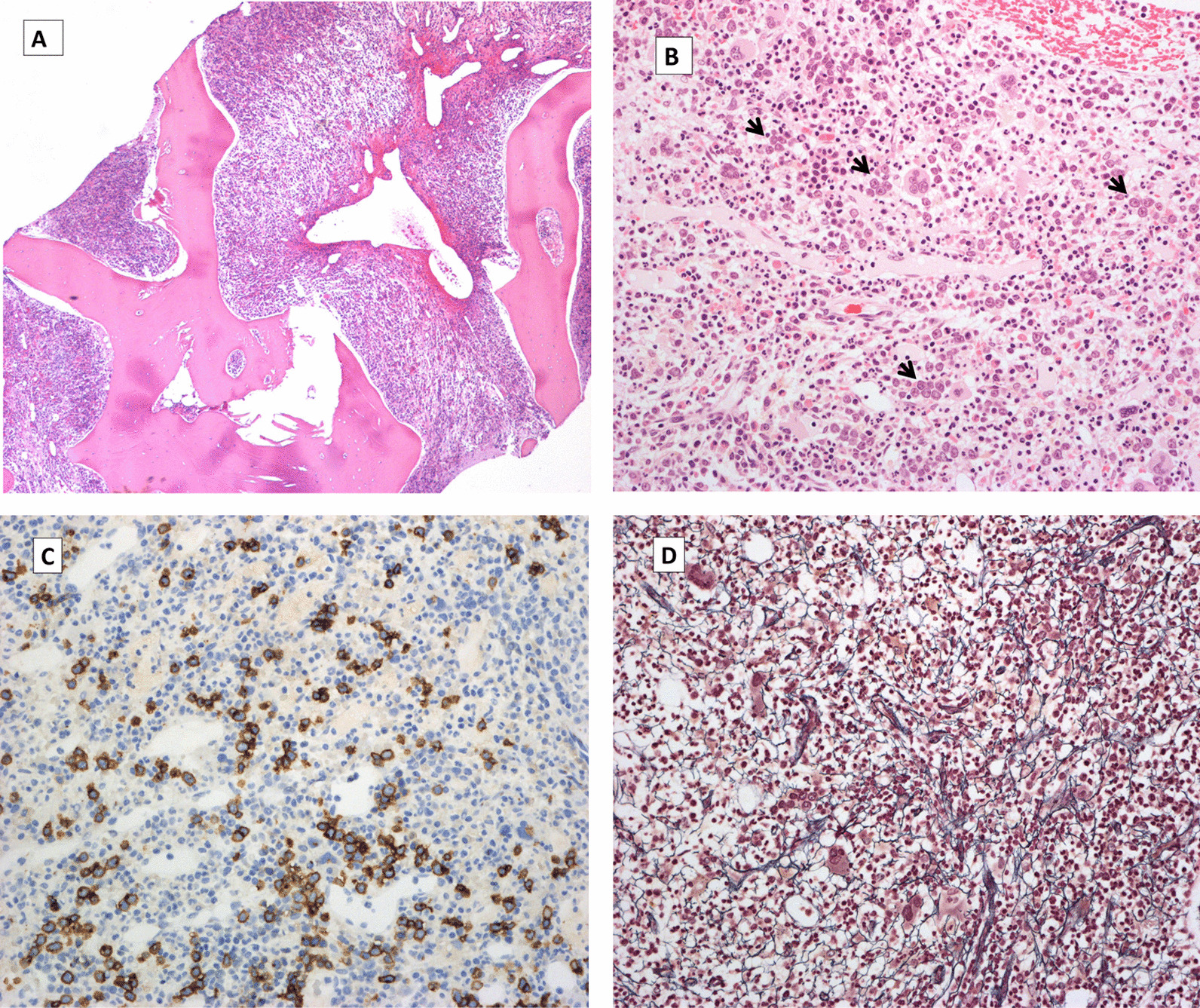
Bone marrow biopsy showing high cellularity (hematoxylin–eosin stain [H&E]; × 40) (**a**). Left-shifted erythropoiesis (arrow heads) (H&E, × 200) (**b**), highlighted by E-cadherin stain (× 200) (**c**). Grade 1–2 fibrosis, Gomori stain (× 200) (**d**)

Without evidence of malignant disease or other acute life-threatening conditions, the patient was observed for four months without any specific therapy. Treatment with natalizumab was stopped, and no other MS-directed therapy was initiated. There was no sign of spontaneous remission, and she remained transfusion dependent. Subsequently, a trephine biopsy was performed. The bone marrow displayed maximal cellularity, with all lineages represented. Erythropoiesis showed a marked left shift. There was reticulin fibrosis of grades 1 to 2 of 3 and a slight T lymphocyte hyperplasia was noted. No features of myeloproliferative neoplasia or myelodysplasia was detected (Fig. 1a–d). This indicated a pathologic process halting the maturation of erythrocytes. Bone marrow aspiration at this time resulted in a dry tap; thus, karyotyping was not performed.

Endogenous erythropoietin was markedly elevated at 652 IU/L. Tests for autoimmune antibodies were negative (anti-nuclear antibodies, anti-DNA antibodies, anti-neutrophil cytoplasmic antibodies MPO and PR3, anti-actin antibodies, anti-M2 antibodies, anti-CCP antibodies). A monoclonal T cell receptor (TCR) gene rearrangement on a polyclonal background was found. Further molecular testing with a myeloid mutation panel was done using Illumina’s TruSight Sequencing Panel, covering 54 commonly mutated genes. No somatic mutations with an allele frequency > 5% were detected. Paroxysmal nocturnal hemoglobinuria was ruled out by flow cytometry.

Seven months after admission, the hemoglobin was still low (8.9 g/dL). A blood smear showed that two thirds of lymphocytes were large granular lymphocytes. Flow cytometry of peripheral blood demonstrated normal levels of T and B lymphocytes and NK cells. The T lymphocytes had a normal CD4/CD8 ratio and a normal phenotype. Approximately 50% of the CD8 + T lymphocytes expressed CD57. The same clonal TCR gene rearrangement was still present. One month later, erythropoiesis showed signs of regeneration, and 9 months after natalizumab was discontinued, the patient had a hemoglobin level of 11.6 g/dL. There was no further need for transfusions, and after nearly one year, the patient had fully recovered. She did not receive natalizumab or any other MS treatment after she was admitted with severe anemia. In total, she received 13 units of packed red blood cells.

## Discussion

The main findings of the diagnostic work-up were hypercellular bone marrow, reticulin fibrosis of grades 1–2, and marked left shift of erythropoiesis. Since the anemia was macrocytic, a deficiency of vitamin B_9_ and B_12_ were considered but was excluded early on. Excessive intake of alcohol is another common cause of macrocytic anemia, but this was not the case in our patient. Liver function tests were normal. Reticulocytosis may give rise to mild macrocytosis, but our patient had a normal number of reticulocytes [7]. Myelodysplastic syndromes (often present with macrocytic anemia, but are uncommon in young adults, with only 0.1 cases per 100 000 person-years below 40 years [8]. Myelodysplastic syndromes were ruled out by the absence of significant dysplastic features and no molecular genetic aberrations. Endoscopic investigation of the GI-tractus was considered not indicated because of no signs and symptoms of macroscopic blood loss, negative fecal occult blood test and no laboratory findings compatible with iron deficiency.

Autoimmune myelofibrosis was also considered but was unlikely due to negative autoantibodies and the characteristics of the bone marrow, with a left shift of erythropoiesis [9]. A low haptoglobin and a modest increase in lactate dehydrogenase indicated hemolysis, and DAT was positive. Anti-IgG negative and anti-C3d positive are DAT signatures typical of cold agglutinin disease, but cold agglutinins were not detected. Hemolytic anemia is a recognized complication of natalizumab. Midaglia et al. and Rivero et al. reported cases of severe autoimmune hemolytic anemia with a similar DAT following treatment with natalizumab [10, 11]. They showed the antibody to be drug-dependent. However, it is also known that 1–15% of hospitalized patients may have a positive DAT without overt signs of hemolysis [12]. The normal amount of reticulocytes is an argument against autoimmune hemolytic anemia in our patient, and the DAT was therefore considered non-significant. DAT repeated 50 months after cessation of natalizumab was weakly positive but negative for both IgG and C3d. We also found clonal TCR gene rearrangement, indicating immunological dysregulation. However, this was still present when our patient had recovered from the anemia, and the clonal TCR gene rearrangement was most likely a confounding finding.

The patient was found to have mild splenomegaly of 15 cm on CT scan. There are multiple causes for splenomegaly including hemolytic anemia and hematologic malignancies like lymphoid neoplasms. Sometimes etiology can´t be found [13]. Since the anemia spontaneously improved, the splenomegaly is likely not a major factor in the pathogenesis, although the positive DAT and signs of a clonal T-cell population could potentially be linked to the enlarged spleen.

Parvovirus B19 infection is another cause of anemia, usually in patients with underlying hemolytic anemia or who are immunocompromised [14]. This possibility was also ruled out from microscopic evaluation of the bone marrow and later from a negative immunohistochemistry for parvovirus on the bone marrow biopsy.

**Fig. 2:**
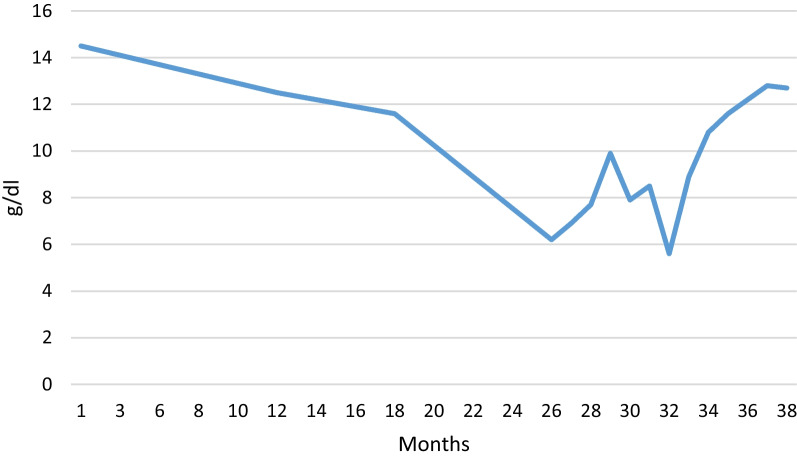
Curve of hemoglobin

From the very beginning, natalizumab was suspected to be the cause of the anemia, and the treatment was stopped upon admitting the patient. She did not receive any other treatment for her MS. Her only additional medication was medroxyprogesterone acetate injections every three months from one year before admission. A close assessment of medical records disclosed a fall in hemoglobin from around 14.5 g/dL to 12.5 g/dL after one year of natalizumab treatment. This development continued, and 27 months after initiation of treatment with natalizumab, hemoglobin was 6.2 g/dL (Fig. 2).

Anemia is reported as a common side effect of natalizumab, occurring with a frequency between 1 and 10%, but severe anemia is rare, affecting 1 of 1000 patients. Anemia is also observed in neonates delivered by women receiving natalizumab during pregnancy. The mechanism of natalizumab-induced anemia is thought to be related to the presence of VLA-4 on the surface of fetal blood precursors [15]. The goal of natalizumab treatment is blocking VLA-4 on lymphocytes, preventing migration through the blood–brain barrier with an intention to reduce inflammation in the central nervous system [1]. Both VLA-4 and VLA-5 are found on erythroblasts and are thought to regulate erythroid differentiation [16]. Adherence of erythroblasts expressing VLA-4 to macrophages expressing VCAM-1 plays an important role in the maturation and proliferation of erythroid precursors [17]. Blockage of VLA-4 can thus contribute to the inhibited maturation of erythroblasts.

There are a few published reports of severe anemia associated with natalizumab [2–6]. These reports are summarized in Table 2 describing the main findings, treatment and duration of anemia. Simone et al. and Seibert et al. separately reported a case of severe anemia following natalizumab treatment with striking similarities to our case: macrocytic anemia and a hypercellular bone marrow with maturation arrest in erythropoiesis and fibrosis [5, 6]. Both patients recovered when natalizumab was discontinued. The authors did not report on DAT in their patients. Two other cases described treatment with corticosteroids alone or in combination with intravenous epoetin alfa [3, 4]. It is uncertain whether this type of treatment can shorten the time to the regeneration of erythropoiesis, although it seems that spontaneous remission will nonetheless occur. Previous reports describe recovery of anemia within two to five months after stopping natalizumab, which could be expected considering the half-life and pharmacokinetics of the drug. Our patient had a complete recovery close to one year after discontinuing natalizumab.

**Table 2:** Reported cases with severe anemia related to natalizumab

Case	Mechanism and main finding described	Treatment for anemia	Duration of natalizumab treatment	Duration of anemia	Author
Present case	Maturation arrest of erythropoiesis, bone marrow fibrosis	RBC-transfusion	27 months	9 months	Hamnvik et al
Case 1	Maturation arrest of erythropoiesis, bone marrow fibrosis	RBC-transfusions	34 months	2–3 months	Simone et al
Case 2	Hyporegenerative anemia	Alfa-epoietin, Corticosteroids, RBC-transfusions	15 months	5 months	Monteleone et al
Case 3	Hyporegenerative anemia	Vitamin B and folic acid, RBC-transfusions	16 months	4 months	Monteleaone et al
Case 4	Hyporegenerative anemia	RBC-transfusions	20 months	5 months	Seibert et al
Case 5	Hyporegenerative anemia, maturation arrest of erythropoiesis, bone marrow fibrosis	Corticosteroids, RBC-transfusions	78 months	3 months	Bachofner et al
Case 6	Drug-dependent, DAT positive hemolytic anemia	Intravenous immunoglobulins, corticosteroids, RBC-transfusions	1 week (1 dose)	2 weeks	Midaglia et al
Case 7	Drug-dependent, DAT positive hemolytic anemia	Intravenous, Immunoglobulins, Corticosteroids, RBC-transfusion	2 months (2 doses)	2–3 weeks	Rivero et al

*DAT* Direct antiglobulin test;* RBC* Red blood cell

The reason for this long-lasting anemia is unclear. The estimated median half-life of natalizumab is around 27 days, with the 95th percentile interval of the terminal half-life ranging from 11.6 to 46.2 days [18, 19]. Therapeutic drug monitoring of natalizumab can be done by measuring serum concentration and α4 integrin saturation [18, 20, 21], and there are large individual variations in serum concentrations [20, 21]. Body weight and anti-natalizumab antibodies have been found to affect both serum concentrations and the clearance of natalizumab; antibodies and high body weight lower the concentration of the drug and increase clearance. Further, natalizumab may still show some binding to α4 integrin even though it is cleared from the plasma [22]. Plavina et al. showed that it takes around 16 weeks before natalizumab is undetectable in the serum, but that some antibodies may be detected on cells up to 28 weeks after interruption [23]. Our patient had no evidence of anti-natalizumab antibodies but had a body mass index of 33.5. Thus, a lower concentration and faster clearance of the drug, resulting in a more rapid resolution of the anemia, may be expected. Muralidharan et al., however, showed that α4 integrin saturation was not significantly affected by body weight [18], and that other factors such as receptor density and turnover contribute to the known variability of α4 integrin saturation. We did not measure drug serum concentration or α4 integrin saturation, which could have been of interest considering the long-lasting anemia.

The fibrosis in the bone marrow in our case and in other cases is of uncertain etiology. Fibrosis is seen in many different conditions other than neoplastic processes and can be reversible. Changes in the bone marrow microenvironment and cellular composition are usually mentioned as some of the mechanisms of fibrosis development [24]. The marked left shift of erythropoiesis, and thus changes in the bone marrow cellular environment, could perhaps stimulate fibrosis.

## Conclusion

This case underscores the possibility of developing a severe anemia during treatment with natalizumab, a recombinant anti-α4β1 integrin antibody. It shows that natalizumab-related anemia can be long lasting, up to a year. The mechanism of this side effect is probably related to the presence and subsequent blocking of α4β1 integrin on erythropoietic precursor cells.

## Data Availability

Not applicable.

## References

[1] von Andrian UH, Engelhardt B (2003). α4 integrins as therapeutic targets in autoimmune disease. N Engl J Med.

[2] Bachofner A (2019). Natalizumab: hyporegenerative anaemia secondary to erythroid maturation arrest: case report. Reactions weekly.

[3] Mansoor S (2020). Natalizumab-induced hyporegenerative anaemia and leukopenia: a case report. Egypt J Neurol Psychiatry Neurosurg.

[4] Monteleone F (2015). Reversible hyporegenerative anemia during natalizumab treatment. Mult Scler.

[5] Seibert JB, Alvarez E (2015). Severe anemia in a patient with multiple sclerosis treated with natalizumab. Neurology.

[6] Simone AM (2014). Severe anemia in a patient with multiple sclerosis treated with natalizumab. Neurology.

[7] Aslinia F, Mazza JJ, Yale SH (2006). Megaloblastic anemia and other causes of macrocytosis. Clin Med Res.

[8] Zeidan AM (2019). Epidemiology of myelodysplastic syndromes: why characterizing the beast is a prerequisite to taming it. Blood Rev.

[9] Vergara-Lluri MEMD (2014). Autoimmune myelofibrosis: an update on morphologic features in 29 cases and review of the literature. Hum Pathol.

[10] Midaglia L, Rodriguez Ruiz M, Muñoz-García D (2012). Severe haematological complications during treatment with natalizumab. Mult Scler.

[11] Rivero NL (2013). Natalizumab: autoimmune haemolytic anaemia: case report. Reactions weekly.

[12] Parker V, Tormey CA (2017). The direct antiglobulin test: indications, interpretation, and pitfalls. Arch Pathol Lab Med.

[13] Pozo AL, Godfrey EM, Bowles KM (2009). Splenomegaly: investigation, diagnosis and management. Blood Rev.

[14] Chisaka H (2003). Parvovirus B19 and the pathogenesis of anaemia. Rev Med Virol.

[15] Godano E (2021). Erythropoietin therapy in a case of neonatal anemia after exposure to natalizumab throughout pregnancy. Ital J Pediatr.

[16] Rosemblatt M (1991). Coexpression of two fibronectin receptors, VLA-4 and VLA-5, by immature human erythroblastic precursor cells. J Clin Invest.

[17] Chasis JA, Mohandas N (2008). Erythroblastic islands: niches for erythropoiesis. Blood.

[18] Muralidharan KK (2017). Population pharmacokinetics and target engagement of natalizumab in patients with multiple sclerosis. J Clin Pharmacol.

[19] EMA, *Tysabri : EPAR - Product Information*. 2022, EMA. p. 76.

[20] Foley JF (2019). Evaluation of natalizumab pharmacokinetics and pharmacodynamics with standard and extended interval dosing. Mult Scler Relat Disord.

[21] Serra López-Matencio JM (2021). Evaluation of Natalizumab pharmacokinetics and pharmacodynamics: toward individualized doses. Front Neurol.

[22] Derfuss T (2017). α4-integrin receptor desaturation and disease activity return after natalizumab cessation. Neurology.

[23] Plavina T (2017). Reversibility of the effects of natalizumab on peripheral immune cell dynamics in MS patients. Neurology.

[24] Kuter DJ (2007). Bone marrow fibrosis: pathophysiology and clinical significance of increased bone marrow stromal fibres. Br J Haematol.

